# Effectiveness of Human Versus Computer-Based Instructions for Exercise on Physical Activity–Related Health Competence in Patients with Hip Osteoarthritis: Randomized Noninferiority Crossover Trial

**DOI:** 10.2196/18233

**Published:** 2020-09-28

**Authors:** Jennifer Durst, Inka Roesel, Gorden Sudeck, Kai Sassenberg, Inga Krauss

**Affiliations:** 1 Department of Sports Medicine University Hospital University of Tuebingen Tuebingen Germany; 2 Interfaculty Research Institute for Sports and Physical Activity University of Tuebingen Tuebingen Germany; 3 Institute for Clinical Epidemiology and Applied Biostatistics University of Tuebingen Tuebingen Germany; 4 Institute of Sports Science University of Tuebingen Tuebingen Germany; 5 Social Processes Lab Leibniz-Institut für Wissensmedien Tuebingen Germany; 6 Faculty of Science University of Tuebingen Tuebingen Germany

**Keywords:** digital app, exercise, movement control, self-efficacy, control competence, mHealth, osteoarthritis, tablet

## Abstract

**Background:**

Hip and knee osteoarthritis is ranked as the 11th highest contributor to global disability. Exercise is a core treatment in osteoarthritis. The model for physical activity–related health competence describes possibilities to empower patients to perform physical exercises in the best possible health-promoting manner while taking into account their own physical condition. Face-to-face supervision is the gold standard for exercise guidance.

**Objective:**

The aim of this study was to evaluate whether instruction and guidance via a digital app is not inferior to supervision by a physiotherapist with regard to movement quality, control competence for physical training, and exercise-specific self-efficacy.

**Methods:**

Patients with clinically diagnosed hip osteoarthritis were recruited via print advertisements, emails and flyers. The intervention consisted of two identical training sessions with one exercise for mobility, two for strength, and one for balance. One session was guided by a physiotherapist and the other was guided by a fully automated tablet computer-based app. Both interventions took place at a university hospital. Outcomes were assessor-rated movement quality, and self-reported questionnaires on exercise-specific self-efficacy and control competence for physical training. Participants were randomly assigned to one of two treatment sequences. One sequence started with the app in the first session followed by the physiotherapist in the second session after a minimum washout phase of 27 days (AP group) and the other sequence occurred in the reverse order (PA group). Noninferiority was defined as a between-treatment effect (gIG)<0.2 in favor of the physiotherapist-guided training, including the upper confidence interval. Participants, assessors, and the statistician were neither blinded to the treatment nor to the treatment sequence.

**Results:**

A total of 54 participants started the first training session (32 women, 22 men; mean age 62.4, SD 8.2 years). The treatment sequence groups were similar in size (PA: n=26; AP: n=28). Seven subjects did not attend the second training session (PA: n=3; AP: n=4). The app was found to be inferior to the physiotherapist in all outcomes considered, except for movement quality of the mobility exercise (gIG –0.13, 95% CI –0.41-0.16). In contrast to the two strengthening exercises in different positions (supine gIG 0.76, 95% CI 0.39-1.13; table gIG 1.19, 95% CI 0.84-1.55), movement quality of the balance exercise was close to noninferiority (gIG 0.15, 95% CI –0.17-0.48). Exercise-specific self-efficacy showed a strong effect in favor of the physiotherapist (gIG 0.84, 95% CI 0.46-1.22). In terms of control competence for physical training, the app was only slightly inferior to the physiotherapist (gIG 0.18, 95% CI –0.14-0.50).

**Conclusions:**

Despite its inferiority in almost all measures of interest, exercise-specific self-efficacy and control competence for physical training did improve in patients who used the digital app. Movement quality was acceptable for exercises that are easy to conduct and instruct. The digital app opens up possibilities as a supplementary tool to support patients in independent home training for less complex exercises; however, it cannot replace a physiotherapist.

**Trial Registration:**

German Clinical Trial Register: DRKS00015759; http://www.drks.de/DRKS00015759

## Introduction

### Background

Osteoarthritis (OA) is characterized by pathological changes of the joint structure and is accompanied by pain and functional limitations. The global burden of hip and knee OA was ranked as the 11th highest contributor to worldwide disability [1]. The prevalence of OA increases with age. In Germany, almost 50% of all women and 30% of all men suffer from OA [2].

The promotion of physical activity in general, and exercise in particular, is highly relevant for patients with OA, as it has been shown to decrease pain and improve physical functioning [3]. Furthermore, physical inactivity is a particular characteristic of critical OA patients, with higher all-cause mortality compared to that of the general population, and is even more pronounced in people with severe walking disabilities [4]. Accordingly, international and national guidelines strongly recommend physical exercise as a core treatment in the management of hip and knee OA [5-8]. However, only a small to moderate proportion of patients with knee and hip OA meet physical activity guidelines [9]. An essential barrier for regular physical exercise could be that people with knee or hip OA suffer from pain at the weight-bearing joints and may further believe that physical activity is not beneficial or is even harmful to their joint [10]. Accordingly, OA patients experience fear of worsening their symptoms through exercise or by executing the exercises incorrectly [11,12].

Supervision seems to be an effective means to promote safe and correct exercise techniques, especially in the initial stages of the disease, and to ensure the right exercise dosage for each individual according to their physical ability and dosage principles from a training science perspective [13,14]. It is recommended that initial supervision and knowledge transfer on activity pacing be guided by a health care provider [7]. However, there is a considerable discrepancy between activity-related recommendations and practice in health care: 63% of patients with hip or knee OA aged 60 years or older that were part of a large German statutory health insurance company were treated with medication, whereas only 41% received physical therapy, including physiotherapy and exercise therapy [15]. Therefore, exercise-related advice from health care providers to community-dwelling people is not yet satisfactory. This is especially true for information related to modifying exercise behavior [16,17].

As a consequence, exploring alternate cost-efficient forms of delivery modes for people with limited access to therapeutic services appears to be indicated [13]. In this context, digital apps could be particularly suitable, since geographical proximity and time synchronicity between a therapist and patient are no longer necessary requirements for therapeutic interventions.

Digital interventions offer various types of interfaces and degrees of supervision. First, blended physiotherapy partially replaces face-to-face appointments with a digital app comprising instructions for graded activity, complementary unsupervised exercises, and behavior-change techniques. The physiotherapist plays an active role in patient interaction with regard to exercise content, as well as in supervision of intervention progress [18]. Second, the intervention can be delivered without the local presence of a physiotherapist, but with permission for regular Skype video sessions and individual support of the home training program in terms of exercise selection, instruction, and dosage adjustment. Exercise videos are also provided [19]. Other interventions still build upon personal supervision, yet without face-to-face contact. Web-based intervention programs are used that provide information, exercise instructions, an online physiotherapist (synchronous and asynchronous chats, and telephone consultations), and education regarding factors of relevance to OA, including lifestyle [20,21]. Third, there are fully automated apps that do not involve any interaction with a real person [22,23]. In the complete absence of personal supervision, a digital app should support the patients’ tasks required for the correct and safe execution of exercises, as well as adequately control physical load and handling of pain as well as possible.

In recent years, a large number of digital exercise interventions have emerged, but there is a lack of studies comparing apps or digital interventions with human-delivered approaches in the field of chronic diseases. Hsu et al [24] compared a person-to-person and a digital-assisted approach for successful aging in older people. The results indicated that healthy behavior improved for the person-to-person group, although not significantly. However, the ability to search for health information improved in the digital-assisted group. Some other studies compared guided (email or telephone) and unguided mental health interventions. Guided interventions were significantly superior to unguided interventions [25]. However, none of the above-mentioned studies included patients with OA. Furthermore, systematic reviews have shown that the theoretical foundation of digital interventions is often not very well-developed [26].

In the present study, we evaluated a fully automated digital app that was developed on the basis of a paper-based home exercise program, which emerged as one of the important features of an evidence-based exercise intervention for patients with hip OA [27,28]. The app was designed to be used without personal supervision and comprises a selection of videos with hip-specific exercises, information on dosage principles, as well as individualized feedback mechanisms (for further details see the Methods section).

### Theoretical Underpinning of Digital Exercise Interventions

With regard to the effectiveness of exercise interventions in general, and for digital interventions in particular, a theory-based approach has been shown to optimize the targeted effects [29-31]. The digital app examined in this study was developed as part of the framework of the model of physical activity-related health competence (PAHCO) [32]. This model follows the general objective of exercise interventions for patients with chronic diseases to promote individual competence for an increasingly independent realization of regular physical exercise [33]. From this health educational perspective, it is particularly important to empower the patients to adequately master the requirements of specific exercises and to enable them to carry out physical exercise in the best possible health-effective and low-risk manner, taking into account their own physical condition.

The PAHCO model considers three subcompetences, each of which specifically helps in coping with demands that arise during the initiation and maintenance of physical exercise in a health-enhancing way. *Movement competence* relates to motor demands and is observable based on high movement quality while performing physical exercises. Movement competence is mainly based on motor abilities and skills but also requires the confidence to accomplish the task with one’s own abilities (corresponding to task self-efficacy [34]). *Control competence* enables people to gear their own physical exercise to optimize health benefits and minimize health risks. It is mainly based on action-related knowledge but also requires the ability to perceive and interpret body signals (eg, to adjust intensities based on muscle soreness). *Self-regulation competence* ensures the required regularity of physical exercise. Thus, in theoretical terms, this subcompetence is closely related to social-cognitive theories of health behavior that address motivational and volitional determinants of exercise behavior (eg, outcome expectancies, behavioral self-efficacy; for an overview see Biddle et al [35]). The subcompetences can be considered as proxies for regular physical exercise, which in turn leads to the desired health benefits [32]. The promotion of these subcompetences should therefore be an important aim of an exercise intervention. Although pain, physical functioning, fear of movement, and self-efficacy have already been the focus of previous studies on the effectiveness of digitally assisted training interventions [23,36,37], there is a lack of studies related to proxies of health outcomes such as movement quality, control competence, and self-regulation.

### Aims and Hypotheses

Based on the aforementioned knowledge deficits, the aim of this crossover study was to evaluate whether exercise instruction and guidance of one training session via a fully automated tablet computer-based app results in comparable benefits in subcompetences of PAHCO in comparison to one session supervised by a physiotherapist in subjects with hip OA. Our hypotheses were as follows: (1) movement quality of exercises guided by the app is not inferior to the movement quality under supervision by a physiotherapist; (2) the effect of the app on control competence is not inferior to the effect of the physiotherapist-guided intervention; and (3) the effects of the app on exercise self-efficacy (as a prerequisite of self-regulation competence) are not inferior to the effects of the physiotherapist-guided intervention.

## Methods

### Study Design

This study was designed as a randomized 2×2 crossover trial. The participants were randomly assigned to one of two exercise treatment sequences with an allocation ratio of 1:1. The AP sequence started with a training session instructed by a fully automated tablet computer-based digital app, followed by an intervention supervised by a physiotherapist, whereas the PA sequence started with the physiotherapist and the second training intervention was conducted with the app. The washout phase between the two interventions was set to range between 3 and 5 weeks. This time period seemed sufficient to washout relevant treatment effects of a single exercise. Ethical approval was obtained from the ethical committee of Tuebingen University Hospital. The study was registered in the German clinical trial register (DRKS00015759).

### Participants

Community-dwelling individuals with diagnosed hip OA were recruited via advertisements in regional newspapers, as well as by emails sent to employees of the University of Tuebingen and Tuebingen University Hospital. In addition, flyers were distributed by orthopedic surgeons and physiotherapists. Interested participants were asked to call staff members for further information. According to ethical guidelines, the subjects were fully informed about the positive effects of exercise therapy for hip OA. They were also informed about the structure and details of the app (ie, feedback mechanisms) and the research questions. The screening for eligibility took place in the context of this phone call. Eligible people were then randomly allocated to one of the two treatment sequences and the dates for the two treatment sessions were scheduled. The treatment sequence was blinded until the first treatment session. Both treatments took place at Tuebingen University Hospital. Inclusion and exclusion criteria for participants are described in Textbox 1.

**Inclusion and exclusion criteria for the noninferiority study.**

- Inclusion criteria
  - 50 years and older
  - self-reported lifetime prevalence of hip osteoarthritis diagnosed by a medical practitioner
  - informed consent to study participation

- Exclusion criteria (general)
  - comorbidities leading to major impairments in everyday life and representing contraindications for physical activities
  - self-reported acute illness
  - significant established osteoporosis requiring treatment, previous spontaneous or low-impact fracture
  - musculoskeletal surgery at the lower extremity within the last 3 months
  - regular use of gait aids (eg, walker, crutch)
  - insufficient German language skills for self-administered questionnaires
  - previous experience in hip exercise groups

- Exclusion criteria (in cases of an artificial joint replacement at the other hip or the knee joints)
  - artificial joint replacement at the knee or hip joint within the last 6 months, with unstable anchoring or with known radiological signs of implant loosening
  - current pain at rest or with activity due to artificial joint replacement
  - luxation as an adverse event of artificial hip joint replacement
  - acute joint inflammation at the knee or hip joint

### Trial Interventions

#### Commonalities of Both Interventions

The interventions used in this study were extracted from an evidence-based 12-week exercise program that was specifically designed for patients with hip OA [28,38,39]. The home-based exercises (2 per week) as well as additional information related to exercise execution, graded exercise dosage, and exercise-related coping with pain are comprehensively described in a book, which also includes training and pain logs [27]. The contents of the book formed the basis for both interventions (physiotherapist and app). Four exemplary exercises of the home training sessions and their instructions were selected from the entire exercise program. The first exercise was related to mobility and movement learning, the second and third were strengthening exercises for the muscles surrounding the hip, and the fourth was a balance task. The training material included elastic rubber bands of different colors, weight cuffs, and soft aero pads. Details on exercise prescription, number of sets, and repetitions are given in Table 1 and Multimedia Appendix 1-4. Participants had to assess their current state of pain prior to each session.

Participants were asked to comment on perceived exertion and OA-related pain after each set using a 10-point Likert scale. The target value for intensity (last row of Table 1) was the same for participants using the app and those supervised by a physiotherapist. In the former case, the app introduced the user to the right intensity. In the latter case, the physiotherapist knew about the adequate intensity and guided the patients to adapt the training intensity if necessary. The instructions for the physiotherapist on how to deal with patients with increasing pain were the same as the algorithm included in the app (first: movement control, second: downgrading of intensity, third: skipping the exercise). A training session, regardless of whether guided by the app or the physiotherapist, lasted between 45 and 60 minutes. The same training area with a size of about 30 m² was used in both cases.

**Table 1:** Details of the exercises.

Detail	Pelvic tilt	Hip abduction	Hip extension	Balance
Name	mobility_seated	strength_supine	strength_table	balance_stance
Position	Seated	Supine	Table-supported stand	Step position
Kit	Stool	Mat, elastic band, pillow	Table, weight cuff, upper body padding	Balance pad
Aim	Pelvic, hip, and lumbar spine mobility; movement learning	Strength endurance	Strength endurance	Balance improvement, fall prevention
Description	Tilting the pelvis back and forth in the sagittal plane	An elastic band is placed below the knees. The feet are set. The knees are slowly tilted outward in the frontal plane	The upper body rests on the table and is supported by the arms. One leg is angled and slowly led back up in the sagittal plane. After adjusting the intensity, the exercise is performed with either an extended leg or an additional weight cuff	Step position both with open and closed eyes as well as with stable and unstable ground
Repetitions	30	20	25 (if exercise is performed with an additional weight cuff, the number of repetitions is reduced to 15)	15 seconds
Sets	2	3	3	6
Intensity	Low, no physical strain	6-7 after the last repetition, still able to perform the exercise correctly	6-7 after the last repetition, still able to perform the exercise correctly	Intensity is related to the difficulty of the balance task and is upgraded as long as the exercise can be performed correctly

#### Specification of Physiotherapist-Guided Exercises

The supervisor was a qualified physiotherapist with 5 years of work experience. She was responsible for (1) introducing exercises, (2) correcting deficient or improper execution of exercises, (3) adjusting intensity, and (4) instructing participants in the case of increasing pain. In addition, it was at the physiotherapist’s judgement whether a participant should skip a simple intensity level and start directly with a more demanding variation.

#### Specification of App-Guided Exercises

The exercises were video-supported. Movement speeds were set using an auditory “click” sound and visually by the training partner in the video (see Multimedia Appendix 1-4). After each set, users were asked to comment on exercise-induced pain and its intensity via a digital visual analog scale on the interface monitor. To ensure a high quality of handling, the tablet was mounted to a holder before the start of the intervention. The participant trained independently and was only guided by the instructions of the app.

#### Characteristics of the App

The app was designed for a 9.7-inch (24.64 cm) Apple iPad and was developed by Ambigate GmbH (Tuebingen, Germany) according to the specifications of the authors. It is not open to the public. In line with the interventional implications of the PAHCO model, the content is basically a combination of practical exercises, cognitive and motor learning, and the processing of personal experience with movement [40]. Based on this, the following 5 different app components were compiled and individual intervention elements were further elaborated with reference to specific theoretical foundations: (1) technical introduction, (2) creation of an individual user profile, (3) pedagogical agent “Emil,” (4) exercise introductions, and (5) feedback-based dose adjustments and further instructions. A detailed list of the app’s components, specific elements, and the theoretical background of interaction principles can be found in Multimedia Appendix 5. An exemplary exercise flow and the underlying simplified algorithm of the software are described in Multimedia Appendix 6.

Videos and acoustic signals were implemented in the software to guide the different exercises and to support the participant during the exercises. The videos can be divided into different categories: (1) instruction video, (2) exercise video, (3) focus video, and (4) video for intensity adjustment. The characteristics of the videos, such as the perspective or benefits of close-ups, were tested in a pilot study during the app development process. The pilot study was conducted on 13 participants aged 50 years and older. This resulted in the implementation of close-ups in both focus videos and exercise videos. In addition, the camera’s perspective was optimized so that starting positions and movements were optimally visible. In addition to the video structure, the pilot study also focused on the choice of adequate actors. The pilot study showed a tendency for gender and age to play only a minor role. It was much more important for the participants that the exercise was performed by the model in a clearly visible and correct manner. Hence, the actors in the videos are middle-aged, a man and a woman, and represent an average of the healthy population.

### Outcome Measures

#### Baseline Data

Sociodemographic, anthropometric, personal, and OA-related variables were used to characterize the sample, including age, gender, educational level, work-related life situation, previous experience with exercise groups, importance of sport throughout life, and technical affinity, which were collected at baseline. In addition, fear of movement, OA-related symptoms, and physical activity in the preceding 4 weeks were collected at the beginning of the first (T0) and second (T1) treatment sessions.

#### Movement Quality

Movement quality was assessed by two independent raters. Both were research assistants with a bachelor degree in exercise science. They were introduced to the scoring procedure of movement quality using a rating sheet including different categories of movement quality for each exercise. The categories described the starting and ending position of all four exercises, as well as the movement sequence for the exercise. The categories are based on the description of the movement order and do not allow for high variance in the response. However, the aim was to query each movement step of the respective exercise. Raters had to classify whether the execution in a category was fulfilled, partly fulfilled, or not fulfilled, and whether all quality criteria points were met throughout all repetitions and the whole movement execution, temporarily, or not at all. The different categories were weighted according to their relevance. The weighting of the individual categories was based on the therapeutic relevance of the respective category for a correct, effective, and safe execution of the movement.

Most of the categories used a 3-point Likert scale with the grades as defined above. If a category could only be fulfilled or not fulfilled, a dichotomous scale was used. The judgments were based on video and audio recordings of the training sessions. Each session was videotaped from two perspectives, so that each movement could be assessed in the appropriate body axis. The raters were allowed to review the videos using the control unit of the video player, if necessary. The values for each exercise were transformed to a scale of 0-100%. All sets were included and averaged across raters. Different scores were calculated: the average value across all categories was defined as the primary outcome for movement quality, and the four average values for each of the exercises were defined as secondary outcomes for movement quality.

#### Exercise-Specific Self-Efficacy

Exercise-specific self-efficacy was measured with a self-assessed questionnaire based on the Multidimensional Self-Efficacy for Exercise Scale [41] before and after each session. The scale ranges from not at all safe (0) to absolutely safe (10). The 9 Likert-like items of the scale can be classified into three subcategories: task, coping, and scheduling. The overall score of exercise-specific self-efficacy was defined as the primary outcome measure of this scale. Overall exercise-specific self-efficacy and its subscales were tested for internal consistency using Cronbach α.

#### Control Competence for Physical Training

The assessment of control competence for physical training was conducted in accordance with the self-rating scale developed by Sudeck and Pfeifer [32]. The participants filled in a questionnaire related to control competence for physical training before and after both training sessions. Six Likert-scale items addressed the application of training-specific knowledge, and using the perception of body signals and perceived exertion to pace and structure exercise and training, targeting either endurance or strength. The Likert scale ranged from “totally disagree” (1) to “totally agree” (4). To further reflect specific demands for patients with hip OA, four additional items were created similar to the existing item set, especially focusing on hip-related exercises. The mean value across all items was used to calculate primary outcome measures. Control competence for physical training was tested for internal consistency using Cronbach α.

### Sample Size

The sample size was predefined to a minimum of N=40 without a sample size calculation.

### Randomization

A computer-generated randomized order list in blocks of 5 entries for each of the 2 treatment sequences (AP and PA) was created prior to the start of the study by the study personnel. Eligible participants were randomly assigned to the sequences at the end of the initial phone call. The study staff member entered the name of the caller into the list consecutively in the order of the calls. The list was visible to the study personnel with no further concealment. If randomized subjects canceled the first treatment session, the randomization slot was opened again and used for the first new incoming call of an eligible subject. The order in which raters assessed the movement quality of each participant was randomized for each rater and test day separately using the internet tool random.org [42].

### Blinding

Participants, assessors (raters), and statisticians were not blinded to the treatment sequence or type of intervention. However, participants did not know which treatment sequence they were assigned to before the start of the first training session. The two raters of movement quality were not blinded to treatment sequence or intervention; however, they were not included in any other part of the study, such as data collection or data analysis.

### Statistical Analysis

#### Baseline Data

Baseline sociodemographic, activity-related, and clinical characteristics are summarized for the overall study population and for each of the two treatment sequences separately (PA, AP). Categorical data are presented as absolute numbers and percentages, and continuous data are presented as mean (SD) or median (IQR), as appropriate. Differences between treatment sequences were compared using Pearson chi-square test for categorical data, unpaired Student *t* test, or Mann-Whitney *U* test. The latter was used if the assumption of normally distributed data was violated.

#### Treatment Effects

This study used a 2×2 crossover design to test for noninferiority of the app versus physiotherapist with respect to the primary and secondary outcome measures as outlined above. The following effects must be considered in a crossover design: direct treatment effect (τ), period effect (π), carryover effect (λ), and random subject effect (nested within sequence of treatment order). Therefore, a mixed-model analysis of variance (ANOVA) was used with the fixed factors treatment (physiotherapist, app), period (T1, T2), and treatment sequence (PA, AP), with the latter indicating potential carryover effects.

Treatment effects were averaged over the levels of period and treatment sequence. They were used to calculate crossover standardized effect sizes comparable to independent group designs that were adjusted for medium sample sizes (Hedges g_IG_) [43]. Two-sided 95% CIs were further calculated for the resulting g_IG_. Positive treatment effects (τ) and effect sizes (g_IG_) favor the conventional treatment physiotherapy.

Noninferiority of the app to physiotherapist-guided treatment was established if the upper limit of the 95% CI of g_IG_ was entirely below the predefined noninferiority margin, Δ=0.2 (g_IG_ + 95% CI **<** 0.2). Determining a margin that defines the largest clinically acceptable difference between two treatments is a critical step of a noninferiority trial and should account for both statistical considerations (ie, estimates based on prior studies and clinical judgement). As there is no established recommended Δ value for a noninferiority trial comparing app-based interventions with conventional physiotherapy sessions and no prior studies with similar primary outcome measures are available (to the best of our knowledge), we chose crossover standardized effect sizes equal or greater to an absolute value of 0.2 to be relevant in accordance with the definition of an effect size of Cohen *d=*0.2 to be small with negligible clinical importance. Effect sizes were further categorized with Cohen *d*=0.5 representing a medium effect size and *d*=0.8 representing a large effect size.

Outcome measures of movement quality were assessed only once during each intervention and were not further adjusted for any variable. Outcome measures of exercise-specific self-efficacy and control competence for physical training were assessed directly prior to and after each treatment for both periods (T1, T2). Post-pre differences (ie, change from baseline) were used as input variables for the mixed-model ANOVA for these measures.

#### Rater Agreement for Movement Quality

Rater agreement was assessed by calculating the percentage of exercise-related movement quality categories in which raters completely agreed. Values were averaged across all sets for each exercise and were considered separately for the app and physiotherapist treatments.

#### Sensitivity Analysis for Outcomes with Carryover Effects

If the mixed-model ANOVA exhibited a significant carryover effect, an additional analysis of treatment effects was conducted for period T1 only with a simple *t* test for unpaired samples. Effect sizes were calculated using Hedges *g*. Two-sided 95% CIs were further calculated for the resulting *g*.

The level of statistical significance was set at the conventional level of α=.05. All data were analyzed using SPSS IBM Version 25 and R version 3.6.1.

## Results

### Participants

**Figure 1:**
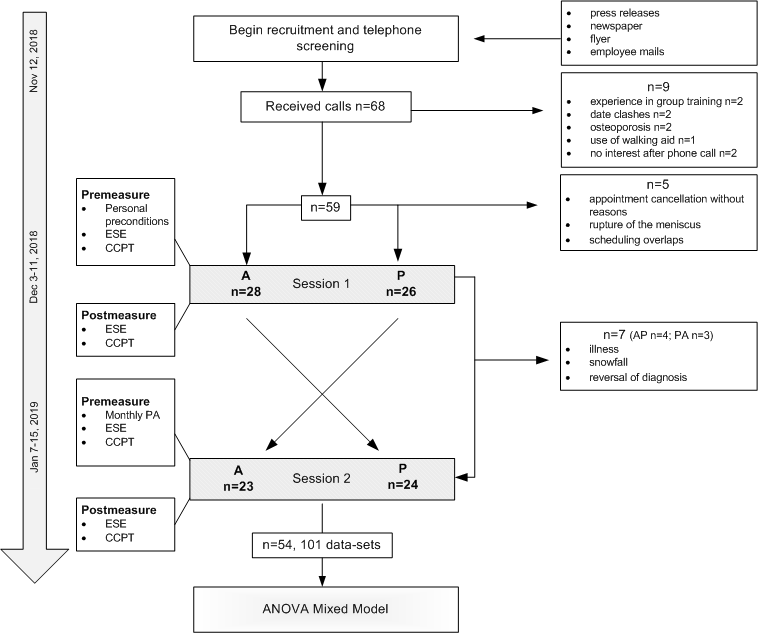
Study flowchart. A: app; P: physiotherapist; AP: app-guided followed by physiotherapist-guided sequence; PA: physiotherapist-guided followed by app-guided sequence; ESE: exercise-specific self-efficacy; CCPT: control competence for physical training; ANOVA: analysis of variance.

Recruitment started in November 2018. Sixty-eight people contacted the study staff by phone, and 59 were deemed eligible and received a follow-up email, including written study information, details on measuring time points, and travel directions. However, five potential participants cancelled the first training appointment due to physical problems or overlapping appointments. Finally, 54 people completed the first training session. Seven participants could not attend the second session. Further details on participants flow are depicted in Figure 1. Data collection started on December 3, 2018 (first patient in) and was completed on January 16, 2019 (last patient out). The individual time period between T1 and T2 ranged between 27 and 42 days, with an average interval of 34.7 days. 

### Baseline Data

Baseline characteristics are presented in Table 2. Subjects in the PA group had a worse baseline condition for hip-related quality of life compared with that of the AP group. Participants in the PA group also had lower values for overall exercise-specific self-efficacy, as well as the cope and schedule subdimensions, in comparison with those of the AP group at baseline. Other baseline characteristics did not differ between participants allocated to the two treatment sequences.

**Table 2:** Baseline data for the complete sample differentiated according to treatment sequence.

Characteristic			Total (N=54)	PA^a^ (n=26)	AP^b^ (n=28)	*P* value
Age (years), mean (SD)			62.4 (8.2)	62.5 (8.0)	62.3 (8.5)	.91
**Gender, n (%)**						.74
	Female		32 (59)	16 (61)	16 (57)	
	Male		22 (41)	10 (39)	12 (43)	
**Education, n (%)**						.19
	Academic education		22 (41)	8 (31)	14 (50)	
	Vocational education		31 (57)	18 (69)	13 (46)	
	No vocational education		1 (2)	0 (0)	1 (4)	
**Employment, n (%)**						.44
	Employed		32 (59)	14 (54)	18 (64)	
	Retired		22 (41)	12 (46)	10 (36)	
Technical affinity^c^, mean (SD)			2.87 (0.4)	2.90 (0.4)	2.84 (0.5)	.65
Previous experience with similar exercises in group sessions^d^, median (IQR)			3.00 (2-3.25)	3.00 (2.0-3.0)	3.00 (2.0-4.0)	.31
Daily activity (minutes of cycling and walking/week), median (IQR)			215 (38-398)	215 (19-349)	225 (60-518)	.49
Sports activity (minutes/week), median (IQR)			209 (59-331)	229 (71-381)	184 (0-308)	.26
Fear of movement^e^, median (IQR)			9.0 (8.0-12.0)	9.0 (8.0-13.3)	9.5 (8.3-10.8)	.84
WOMAC^f^ pain, mean (SD)			31.4 (16.0)	31.4 (16.2)	31.4 (16.1)	.99
**HOOS^g^**						
	Pain, mean (SD)		62.7 (15.5)	62.7 (16.2)	62.7 (15.1)	.99
	Symptoms, mean (SD)		57.8 (17.6)	58.4 (16.4)	57.3 (19.4)	.83
	ADL^h^, median (IQR)		66.9 (54.4-82.7)	64.7 (54.4-77.9)	72.8 (55.2-86.8)	.26
	Sport recreation, mean (SD)		54.2 (22.7)	49.0 (21.7)	58.9 (23.0)	.11
	QL^i^, median (IQR)		43.8 (29.7-62.5)	31.3 (25.0-50.0)	50.0 (37.5-62.5)	.03
**ESE^j^**						
	Overall, mean (SD)		6.13 (1.4)	5.70 (1.1)	6.52 (1.5)	.03
	Task, mean (SD)		6.04 (1.6)	5.85 (1.3)	6.21 (1.8)	.40
	Cope, mean (SD)		5.53 (2.0)	4.88 (1.6)	6.13 (2.1)	.02
	Schedule, median (IQR)		6.83 (5.7-8.1)	6.0 (5.7-7.3)	7.33 (6.3-8.9)	.02
CCPT^k^, mean (SD)			2.67 (0.6)	2.59 (0.5)	2.74 (0.6)	.35

^a^PA: physiotherapist-guided followed by app-guided sequence.

^b^AP: app-guided followed by physiotherapist-guided sequence.

^c^Scored on a 5-point scale from 1 (not true at all) to 5 (fully true); n=3 missing values.

^d^Scored on a 5-point scale from 1 (substantial experience) to 5 (minimal experience).

^e^Scored from 6 (no fear) to 24 (extreme fear).

^f^WOMAC: Western Ontario and McMaster Universities Osteoarthritis Index; pain subscale transformed values from 0 (no pain) to 100 (extreme pain).

^g^HOOS: Hip Disability and Osteoarthritis Outcome Score; transformed values from 0 (extreme impairment) to 100 (no impairment).

^h^ADL: activities of daily living.

^i^QL: hip-related quality of life.

^j^ESE: exercise-specific self-efficacy; rated on a scale from 0 (not at all safe) to 10 (absolutely safe).

^k^CCPT: control competence for physical training; scored on a scale from 1 (totally disagree) to 4 (totally agree).

### Treatment Effects

#### Movement Quality

**Figure 2:**
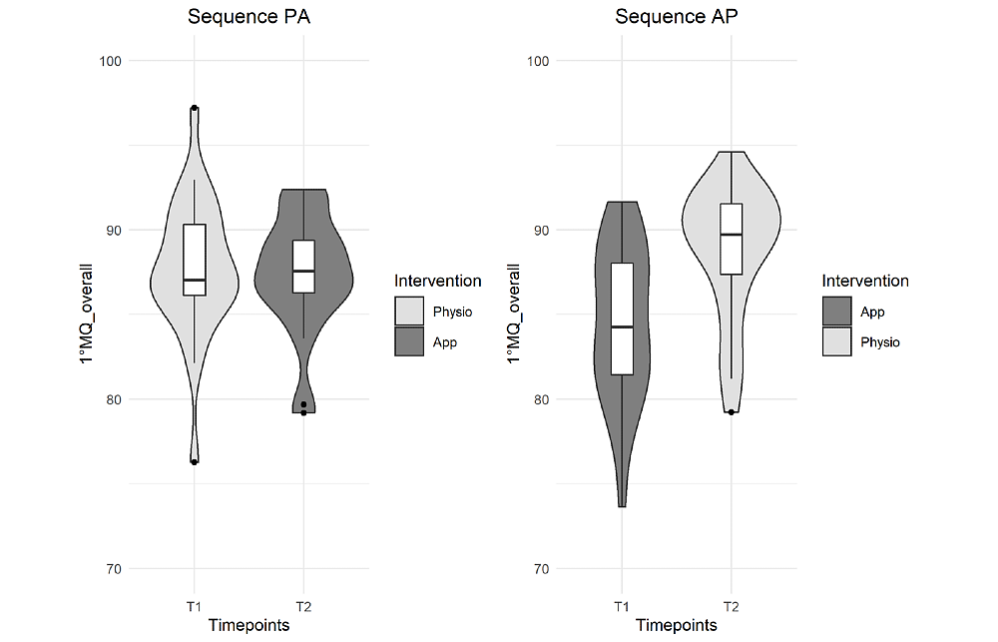
Violin plots (mirrored estimated kernel density plot on each side of the boxplot, tails are trimmed to the range of the data) to visualize the distribution of movement quality (MQ) depending on the treatment sequence and type of intervention. AP: app-guided followed by physiotherapist-guided sequence; PA: physiotherapist-guided followed by app-guided sequence.

**Figure 3:**
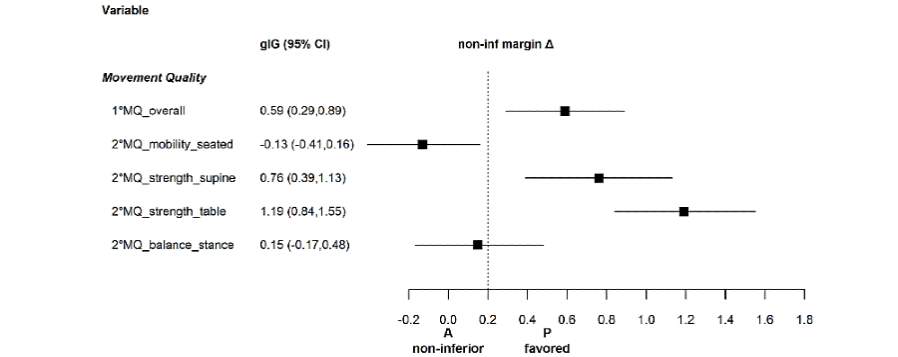
Effect sizes, gIG (95% CI), related to the noninferiority margin for movement quality (MQ).

Results for movement quality are summarized in Table 3 and in Figures 2 and 3. The app was inferior to the physiotherapist in the primary outcome of overall movement quality as well as in all individual exercises, except for the mobility exercise. In contrast to the large effect sizes for the strengthening exercises (supine and table), the effect size for movement quality of the balance exercise was <0.2. However, the 95% CIs exceeded the noninferiority margin.

**Table 3:** Effects of treatment on movement quality (MQ).

Outcome^a^	Estimated mean (95% CI)		Analysis of variance mixed model			Effect size, g_IG_^b^ (95% CI)		ni^c^
	Physiotherapist	App	π^d^ (*P* value)	λ^e^ (*P* value)	τ_d_^f^ (95% CI)			
Primary: MQ_overall	88.3 (87.2-89.5)	85.9 (84.8-87.0)	.002	.37	2.41 (1.21-3.61)		0.59 (0.29-0.89)	0
Secondary: MQ_mobility_seated	83.5 (80.7-86.3)	84.8 (82.0-87.6)	.01	.86	–1.27 (–4.27-1.72)		–0.13 (–0.41-0.16)	1
Secondary: MQ_strength_supine	91.6 (90.2-93.2)	87.9 (86.5-89.2)	.12	.80	3.76 (2.01-5.50)		0.75 (0.39-1.13)	0
Secondary: MQ_strength_table	87.7 (86.2-89.1)	81.4 (80.0-82.8)	.003	.04	6.25 (4.79-7.72)		1.19 (0.84-1.55)	0
Secondary: MQ_balance_stance	90.5 (89.1-91.9)	89.7 (88.3-91.1)	.58	.28	0.78 (–0.93-2.49)		0.15 (–0.17-0.48)	0

^a^ Movement quality (MQ) with 0-100% of quality criteria points fulfilled.

^b^Hedges g_IG._

^c^ni: noninferiority for app (“1” if g_IG_ + 95% CI<0.2; else “0”).

^d^π: period effect.

^e^λ: carryover effect.

^f^τ_d_: treatment effect differences averaged over the levels of period and sequence; positive values indicate a beneficial effect for the physiotherapist.

The overall movement quality score, as well as the movement quality scores for the mobility exercise and strength exercise (table), were significantly better in period 2 (T2) than in period 1 (T1). A statistically significant carryover effect was detected in favor of treatment sequence PA for the movement quality of the table strength exercise.

#### Rater Agreement for Movement Quality

Total agreement between the two raters varied between 79% and 91%. Apart from movement quality for seated mobility, agreement was 3-5% better with the physiotherapist. The highest rater agreement of the assessors was found for the strength exercise in the supine position. Detailed results for movement quality rater agreement are shown in Multimedia Appendix 7.

The results of sensitivity analysis after excluding the movement quality rating categories with unsatisfactory interrater reliability are shown in Multimedia Appendix 8.

#### Exercise-Specific Self-Efficacy

**Figure 4:**
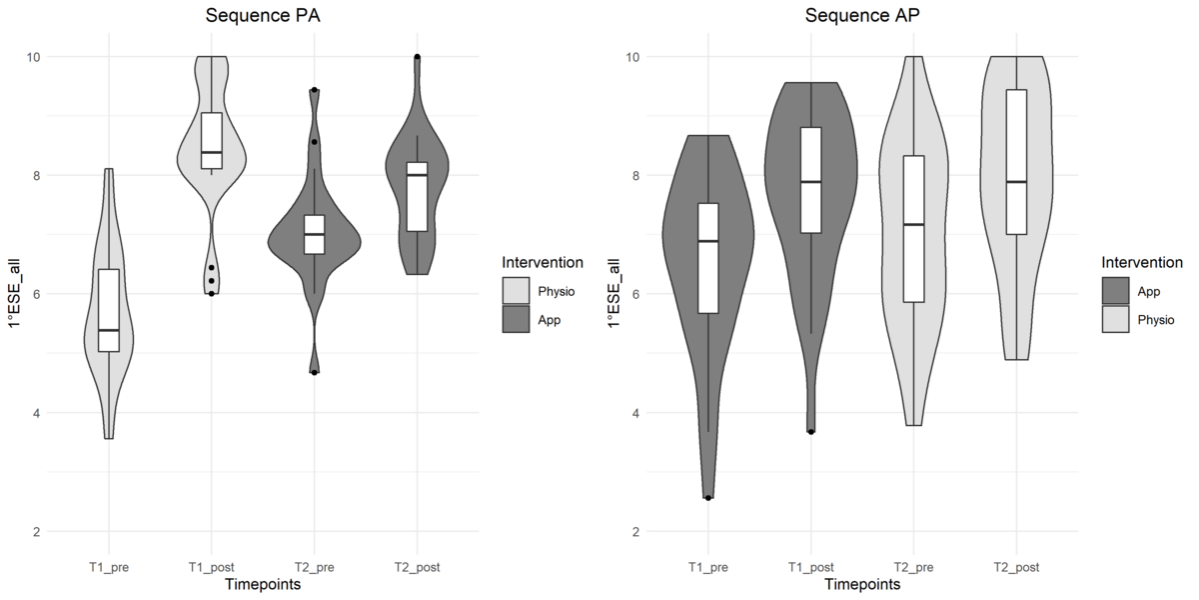
Violin plots (mirrored estimated kernel density plot on each side of the boxplot, tails are trimmed to the range of the data) for visualization of distribution of exercise-specific self-efficacy (ESE) depending on treatment sequence and type of intervention. ESE values range from 0 (not at all safe) to 10 (absolutely safe). AP: app-guided followed by physiotherapist-guided sequence; PA: physiotherapist-guided followed by app-guided sequence.

**Figure 5:**
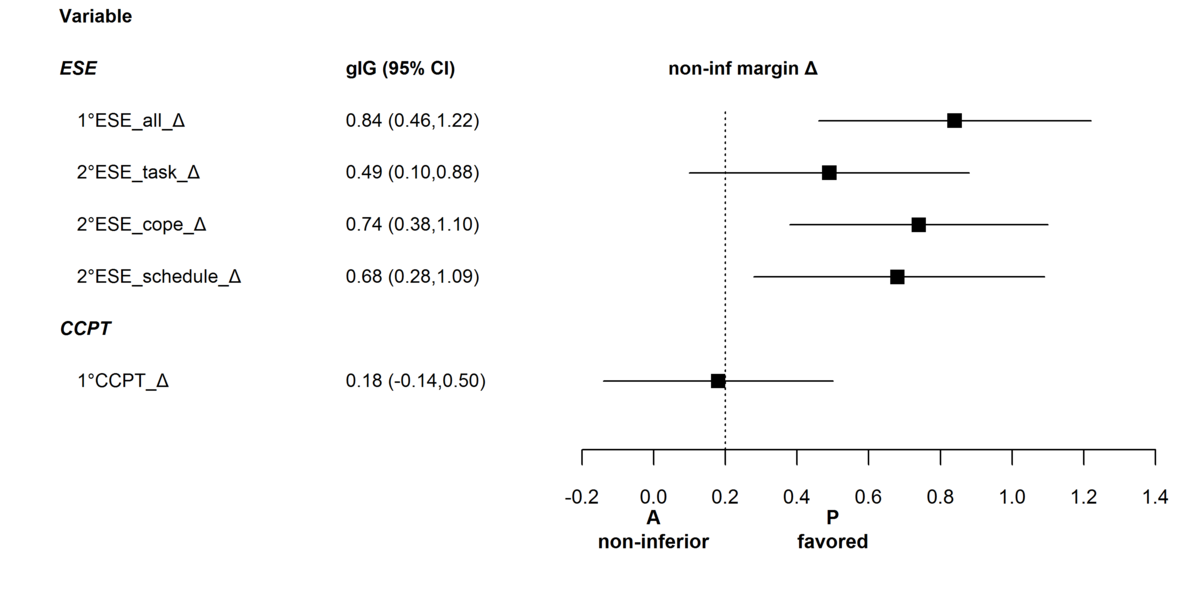
Effect sizes, gIG (95% CI), related to the noninferiority margin for exercise-specific self-efficacy (ESE, overall and with the subdimensions task, cope, and schedule) and control competence for physical training (CCPT). A: app; P: physiotherapist.

The results for exercise-specific self-efficacy are shown in Table 4 and in Figures 4 and 5. The outcomes increased after the intervention in both groups. However, the app was inferior to the physiotherapist.

Overall exercise-specific self-efficacy showed a large effect of the physiotherapist versus the app. Medium effects were found in the exercise-specific self-efficacy subcategories coping and schedule, and a small effect was seen in the subcategory task (Table 4). All measures for exercise-specific self-efficacy showed statistically significant period and carryover effects: intervention effects (post-pre differences) were larger in period 1 compared to those in period 2, and carryover effects were measured in favor of treatment sequence PA. Three of the four scales showed excellent to good scale consistency, with Cronbach α values between .89 and .91. The task subscale also showed an acceptable consistency value with Cronbach α of .79.

**Table 4:** Effects of treatment on exercise-specific self-efficacy (ESE) and control competence for physical training (CCPT) (N=54).

Variable	Estimated mean (95% CI)		Analysis of variance mixed model				Effect size, g_IG_^a^ (95% CI)		ni^b^		Cronbach α	
	Physiotherapist	App		π^c^ (*P* value)	λ^d^ (*P* value)	τ_d_^e^ (95% CI)						
Primary: ESE^f^_all_ Δ	1.85 (1.56-2.14)	0.95 (0.67-1.24)		<.001	.002	0.90 (0.52-1.27)		0.84 (0.46-1.22)		0		.89
Secondary: ESE_task_ Δ	1.96 (1.55-2.37)	1.23 (0.83-1.63)		<.001	.03	0.73 (0.13-1.33)		0.49 (0.10-0.88)		0		.79
Secondary: ESE_cope_ Δ	2.16 (1.75-2.57)	1.06 (0.67-1.46)		<.001	.03	1.10 (0.60-1.60)		0.74 (0.38-1.10)		0		.89
Secondary: ESE_schedule_ Δ	1.41 (1.07-1.75)	0.57 (0.23-0.90)		<.001	.001	0.84 (0.36-1.33)		0.68 (0.28-1.09)		0		.91
Primary: CCPT^g^_ Δ	0.31 (0.17-0.45)	0.22 (0.08-0.36)		.25	.36	0.09 (–0.08-0.26)		0.18 (–0.14-0.50)		0		.94

^a^Hedges g_IG._

^b^ni: noninferiority for app (“1” if g_IG_ + 95% CI<0.2; else “0”).

^c^π: period effect.

^d^λ: carryover effect.

^e^τ_d_: treatment effect differences averaged over the levels of period and sequence; positive values indicate a beneficial effect for physiotherapist.

^f^ESE: exercise-specific self-efficacy; rated on a scale of 0 (not at all safe) to 10 (absolutely safe).

^g^CCPT: control competence for physical training; rated on a scale of 1 (totally disagree) to 4 (totally agree).

#### Control Competence for Physical Training

**Figure 6:**
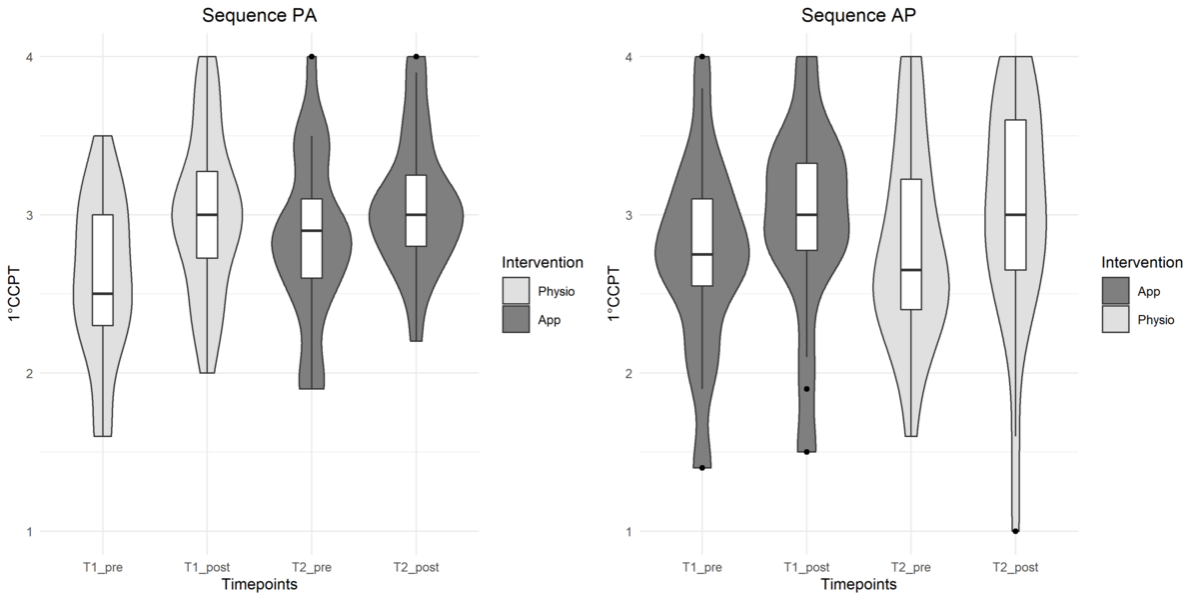
Violin plots (mirrored estimated kernel density plot on each side of the boxplot, tails are trimmed to the range of the data) for visualization of distribution of control competence for physical training (CCPT) depending on treatment sequence and type of intervention. CCPT values range from 1 (totally disagree) to 4 (totally agree). AP: app followed by physiotherapist sequence; PA: physiotherapist followed by app sequence.

As shown in Table 4 and Figures 5 and 6, the outcomes for control competence for physical training increased after the intervention in both groups. However, the app was inferior to the physiotherapist. The effect size was <0.2; however, the 95% CI exceeded the noninferiority margin (Table 4). The scale consistency was excellent with a Cronbach α value of .94.

#### Sensitivity Analysis for Outcomes with Carryover Effects

Outcomes with significant carryover effects were additionally analyzed for period 1 only, and are represented in Table 5. The app was inferior to physiotherapy, with large effect sizes in favor of physiotherapy for all analyzed variables.

**Table 5:** Carryover effects for period 1 only (Student t test for unpaired samples).

Variable	Mean (SD) change from baseline		*t* test, τ_d_^a^ (95% CI)	Effect size, *g*^b^ (95% CI)	ni^c^
	Physiotherapist (n=26)	App (n=28)			
					
Primary: ESE^d^_all_Δ	2.77 (1.1)	1.15 (1.4)	1.62 (0.94-2.31)	1.27 (0.68-1.86)	0
Secondary: ESE_task_Δ	2.96 (1.4)	1.62 (2.0)	1.34 (0.39-2.29)	0.76 (0.20-1.32)	0
Secondary: ESE_cope_Δ	3.10 (1.8)	1.18 (1.6)	1.92 (1.01-2.83)	1.14 (0.56-1.72)	0
Secondary: ESE_schedule_Δ	2.26 (1.3)	0.64 (1.6)	1.62 (0.83-2.40)	1.10 (0.52-1.68)	0
Secondary: MQ^e^_strength_table	87. 8 (4.8)	79.0 (7.2)	8.7 (5.38, 12.10)	1.4 (0.79-2.00)	0

^a^τ_d_: treatment effect differences averaged over the levels of period and sequence; positive values indicate a beneficial effect for physiotherapist.

^b^Hedges *g*_._

^c^ni: noninferiority for app (“1” if g_IG_ + 95% CI<0.2; else “0”).

^d^ESE: exercise-specific self-efficacy; values ranging from 0 (not at all safe) to 10 (absolutely safe).

^e^MQ: movement quality.

#### Harms

No harms or unintended effects occurred during the study.

## Discussion

### Principal Findings

Digital home training programs that help people support their training routines are urgently needed in the current world of decreasing physical activity. This is particularly true for patients suffering from OA. It is well known that exercises are efficient to decrease pain and increase physical functioning in OA [3,5-8]. To enable patients to train in a health-promoting and low-risk range, the PAHCO model provides a breakdown of subcompetences, each of which helps in the long-term realization of health-effective exercise [32,33]. A main interventional implication of the PAHCO model is to combine exercise practice, transfer of knowledge, and processing of personal experience of movement [40].

To the best of our knowledge, there is no app specifically designed for hip OA patients that combines the transfer of knowledge, exercise instructions, and processing of personal experience with movement. Furthermore, there are no studies comparing the effectiveness of these interventions provided by humans and apps. Therefore, the aim of this study was to investigate whether digital exercise instruction and guidance leads to benefits in PAHCO’s subcompetences in a manner that is comparable to physiotherapist care for patients with hip OA.

Movement quality of exercise execution was one important outcome in our study. Exercising in groups of two, in which the partner is alternately practicing and observing, shows important effects on learning success, especially for practical implementation with patients [44-47]. In our study, the physiotherapist served as an active corrective, but less as a role model, because she did not actively execute the exercises. In contrast, the app provides permanent illustrations of how to conduct the exercises via the virtual training partner and additional acoustic signals. The exercises selected for the study differed in terms of their starting position, used musculature, and complexity. Compared to the other two exercises, the two strength exercises were characterized by a more error-prone starting position and execution of movement, whereas the mobility exercise and balance tasks were simpler. Movement quality was inferior in the training session with the app compared with that guided by the physiotherapist, except for the mobility exercise. The difference between the physiotherapist and app for the balance exercise was smaller than the critical effect size value of 0.2, and the criterion for noninferiority was only missed because the 95% CIs exceeded the noninferiority margin. The effects of exercise for mobility and balance in the intervention with the app were close to those with the physiotherapist. However, both strength exercises showed large effects in favor of the physiotherapist-assisted intervention. It can therefore be concluded that not all exercises with different focuses and difficulties are suitable for being guided by a training app only. To avoid comprehension difficulties and poor movement quality, as well as potentially related impairments of training efficacy and risk of harm, we recommend that patients receive supervised instructions when initiating training, especially for more complex exercises. This is in line with results of other investigations, in which initial face-to-face contact with individuals before a new intervention showed positive changes in physical activity outcomes. This is also recommended in guidelines for the nonpharmacological treatment of OA [7,48].

Self-efficacy is an important key to modifying behavior [37]. People with high self-efficacy set higher goals, invest more effort into the pursuit of their goals, and will be more likely to keep trying when barriers and setbacks arise [49]. Self-efficacy increases when the individual receives positive feedback after successfully completing a task [50]. The results from this study revealed that the app is inferior to a physiotherapist in outcomes related to exercise-specific self-efficacy. In particular, the overall scale for exercise-specific self-efficacy as well as its subscales coping and scheduling showed large effects in favor of the physiotherapist. Coping is related to barriers for carrying out exercises, such as “I am able to exercise when I lack energy” or “…don’t feel well,” and scheduling is related to the ability to integrate exercises into daily routines. It can be concluded that face-to-face supervision of a single training session by a physiotherapist cannot be equally replaced by a digital app with a pedagogical agent and video-based training instructions. This was confirmed by Danbjørg et al [12], who reported that the participants missed personal contact with the physiotherapist. However, it should not be disregarded that self-efficacy increased even after training with the app. Litman et al [51] also reported an improvement in self-efficacy using digital apps, but also regarded this type of intervention as a complementary and supporting measure.

We found carryover effects for all outcomes related to the self-administered exercise-specific self-efficacy score, but only for one movement quality outcome. The subjective confidence of being able to show a desired behavior seems to be influenced by a personal supervisor much more than the objective ability to perform a movement. This finding underlines the importance of using both subjective and objective outcome measures if PAHCO is to be improved by a special intervention.

The effect size for control competence for physical training in our study was smaller than the critical value of 0.2, and the criterion for noninferiority was only missed because of the 95% CI exceeding the noninferiority margin. Control competence for physical training is quantified using items that are directly related to the competence to control training intensity in the required way (ie, “I know how I can best increase my strength in the leg and hip area with physical training” or “I am able to adjust my training effort well to my physical condition”). These items are comparable to the exercise-specific self-efficacy subscale task to some extent, with items such as “…I am certain that I will be able to carry out exercises with the right technique.” The app was inferior to the physiotherapist in this subscale. However, only a small effect size was obtained in comparison to all other exercise-specific self-efficacy scales that showed medium or large effect sizes.

The characteristics of our sample are typical characteristics of OA patients, indicating the good generalizability of the findings. The average age and the gender distribution correspond to the risk profile of the disease [52]. The average Western Ontario and McMaster Universities Osteoarthritis Index (WOMAC) pain score attests a moderate hip OA [53]. However, the fear of movement among our participants was relatively low compared to that of other OA samples [11]. Overall baseline values further indicate that the study sample was physically active according to physical activity guidelines, and had a positive attitude toward technology [54]. These criteria may restrict the external validity of the results to a comparable population with respect to fear of movement, physical activity status, and technical affinity.

No adverse events were reported for any of the interventions. However, safety aspects are extremely relevant for fully automated computer-based interventions as there is no health care professional controlling for nonphysiological or even harmful execution of exercises. The app tested in this study used personalized closed feedback loops to adapt exercise instructions and dosage according to the user’s feedback on pain and physical exhaustion. Nonetheless, it has been shown that movement quality was inferior by using the app when it comes to more complex exercises. Future studies with longer intervention periods should therefore evaluate if minor movement competence goes along with higher pain levels, more adverse events, or poorer health outcomes. Proof of safety and medical benefit or patient-relevant structural and process improvements are mandatory aspects for approval of an “app on prescription” in Germany [55].

### Limitations

Results of this study show ceiling effects for the variables self-efficacy and control competence. The study population already had very good values at baseline, thereby reducing the possibility for change. To be able to assess effects in a more differentiated way, samples of future studies should have lower initial values in this outcome dimension. To investigate outcome effects in a more vulnerable population, further research should also focus on a sample that is less physically active, has more severe symptoms, feels greater barriers to technology, and has a greater fear of movement. This limitation and the above-mentioned restrictions with regard to the external validity of the study results may be caused by a potential recruiting bias, which may have affected the outcomes as well. Subjects were fully informed about the rationale and aim of the study in the context of recruitment and inclusion. It therefore cannot be ruled out that this information may have had an effect on user self-selection and expectation, and may have therefore biased the results.

This study also has some methodological issues that should be discussed. Five outcomes of the study showed carryover effects in favor of the physiotherapist, four of which were related to exercise-specific self-efficacy. Although a washout phase with a minimum of 3 weeks was conducted between training sessions, this phase was not long enough to eliminate positive treatment effects. The session with the physiotherapist induced long-lasting effects that sustained during the washout phase. As a consequence, a sensitivity analysis was conducted and results of this sensitivity analysis led to similar but even more pronounced statements related to the inferiority of the app versus the physiotherapist. We are aware that the practice of analyzing data from the first study period as if it had been obtained from a conventional parallel-group design has been shown to be potentially strongly anticonservative [56]. Yet, no conclusive answer is provided by the literature on how to proceed if analyses yield significant carryover differences. Future studies should use a parallel-group design or significantly extend the washout phase to an adequate length to eliminate carryover [57]. However, the results of our study indicate that the latter seems to be difficult to implement in studies with exercise interventions. A complete washout was also not desired in our study.

Exercise-specific self-efficacy and control competence for physical training were assessed prior to and after each intervention, and change from baseline was used as a dependent variable for each intervention. There is little agreement in the current literature as to whether or how to introduce period-specific baseline measurements into the model. The use of change from baseline is discouraged due to its poor type I error rate control and lower power than other methods, yet under the assumption of no carryover [58]. Nevertheless, change from baseline was applied in our study, as it is the most simplistic way to analyze the data, and other methods face similar problems in the presence of carryover effects [59].

Aside from carryover effects, period effects were present in almost all measures. Effects of differences between pre and post values of the training session were larger in period 1, whereas movement quality assessed while exercising was better in period 2. This seems conclusive, as the possibility of improvement within one training session may have a saturation effect partially due to ceiling effects, whereas the better movement quality in period 2 may be related to a learning effect on exercise execution from period 1. As period effects do not affect comparison between groups, this limitation does not seem crucial for the interpretation of the results.

### Conclusion

Despite the absence of noninferiority to the physiotherapist in almost all measures of interest, exercise-specific self-efficacy and control competence for physical training also improve using an app, and movement quality is acceptable for exercises that are easy to perform. However, relevant differences in movement quality are present in challenging tasks. The digital app therefore opens up possibilities to take on the role of a supplementary tool to support the patient in independent home training for less complex exercises. Nevertheless, it cannot replace a physiotherapist with an equivalent effect.

## Appendix

Multimedia Appendix 1 Screenshot of the exercise video “Pelvic tilt.”.

Multimedia Appendix 2 Screenshot of the exercise video “Strengthening of the hip abductors.".

Multimedia Appendix 3 Screenshot of the exercise video “Strengthening of hip extensors.”.

Multimedia Appendix 4 Screenshot of the exercise video "Balance task.".

Multimedia Appendix 5 App components and theoretical background of the interaction principles.

Multimedia Appendix 6 Exemplary decision path of the software algorithm based on the exercise to strengthen the hip abductors.

Multimedia Appendix 7 Rater agreement for movement quality, averaged across all categories and sets for each exercise and intervention in percent. P: physiotherapist; A: app.

Multimedia Appendix 8 Sensitivity analysis for movement quality after excluding categories with poor interrater reliability.

## Abbreviations

ANOVA: analysis of variance
AP: app-guided followed by physiotherapist-guided sequence
OA: osteoarthritis
PA: physiotherapist-guided followed by app-guided sequence
PAHCO: physical activity-related health competence
