# Supplementary figures and images for: Effectiveness of Human Versus Computer-Based Instructions for Exercise on Physical Activity–Related Health Competence in Patients with Hip Osteoarthritis: Randomized Noninferiority Crossover Trial
Source: J Med Internet Res. 2020 Sep 28;22(9):e18233. doi: 10.2196/18233 (PMC7551118; doi:10.2196/18233)

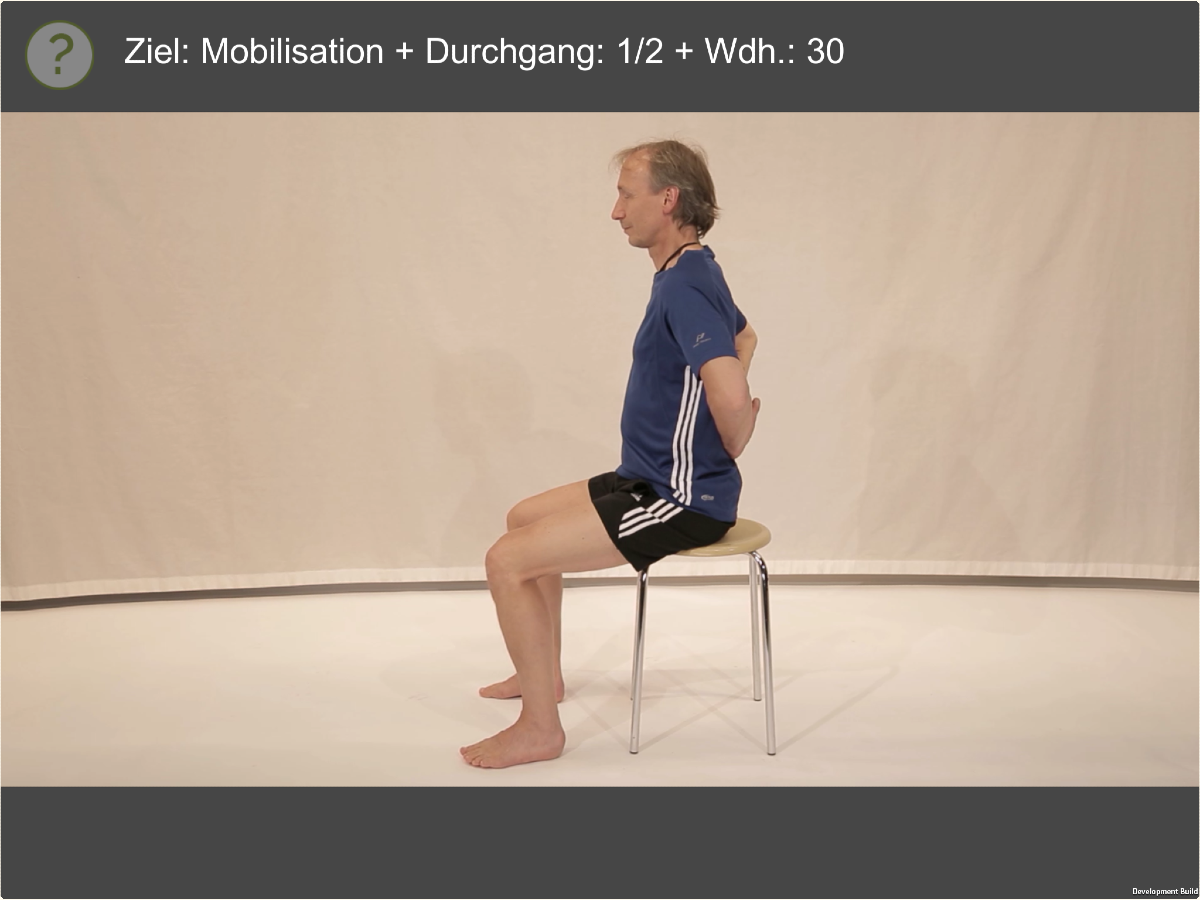

Supplement: Multimedia Appendix 1 [file jmir_v22i9e18233_app1.png]

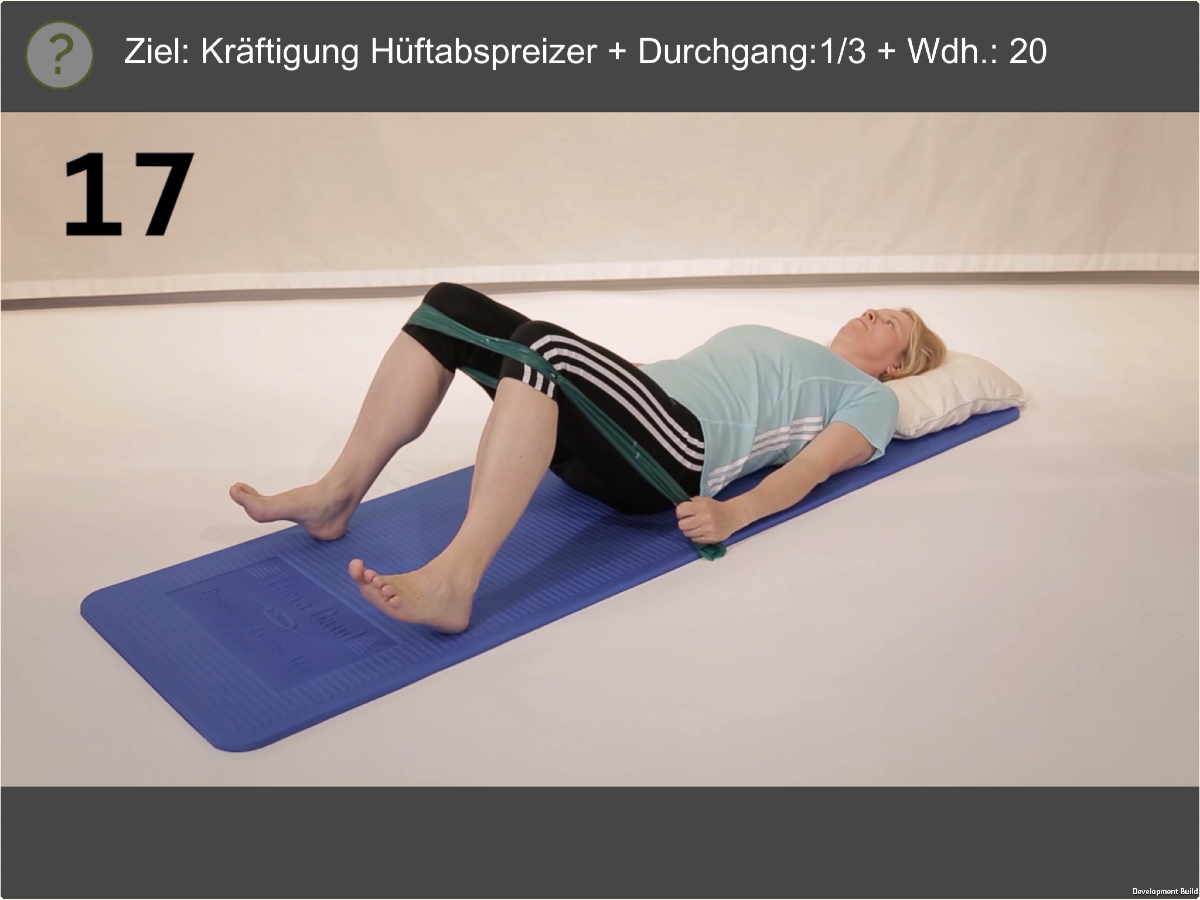

Supplement: Multimedia Appendix 2 [file jmir_v22i9e18233_app2.png]

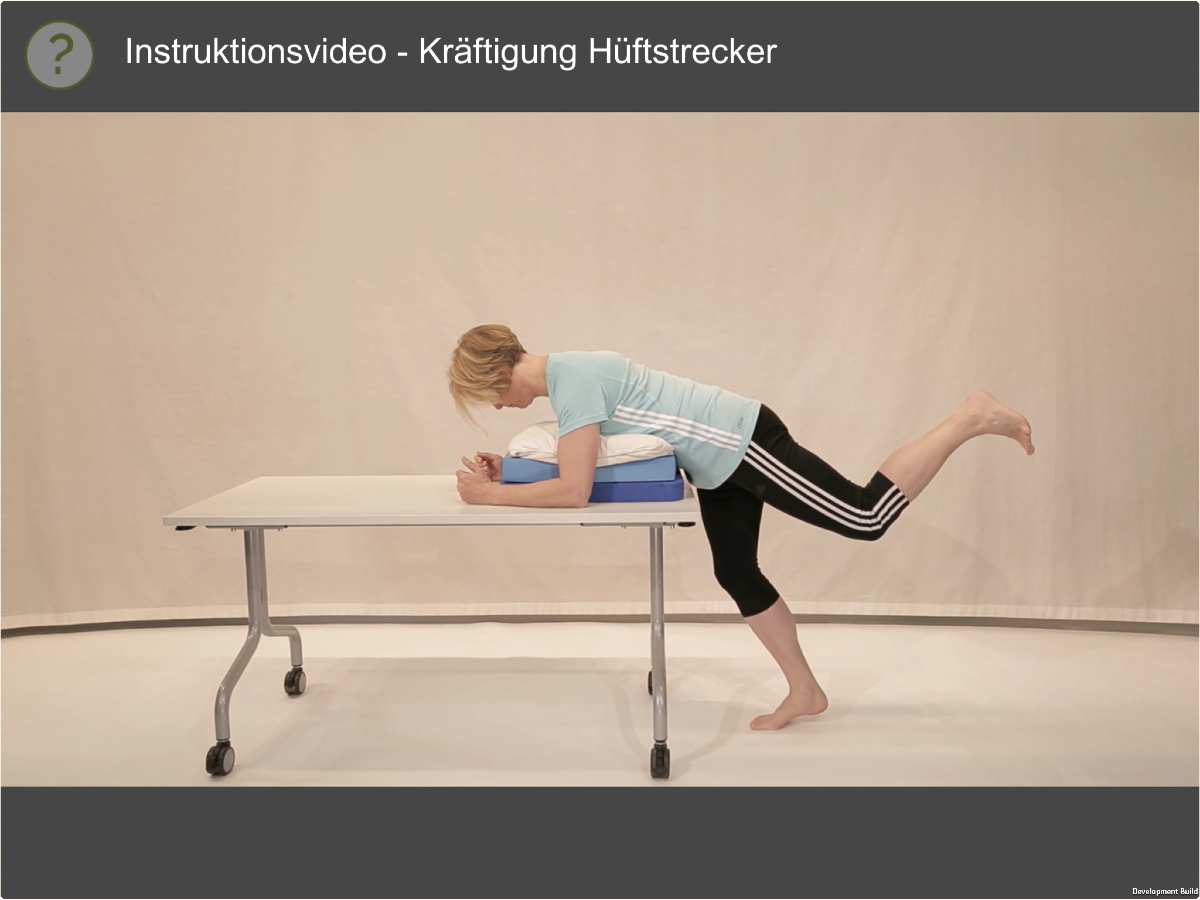

Supplement: Multimedia Appendix 3 [file jmir_v22i9e18233_app3.png]

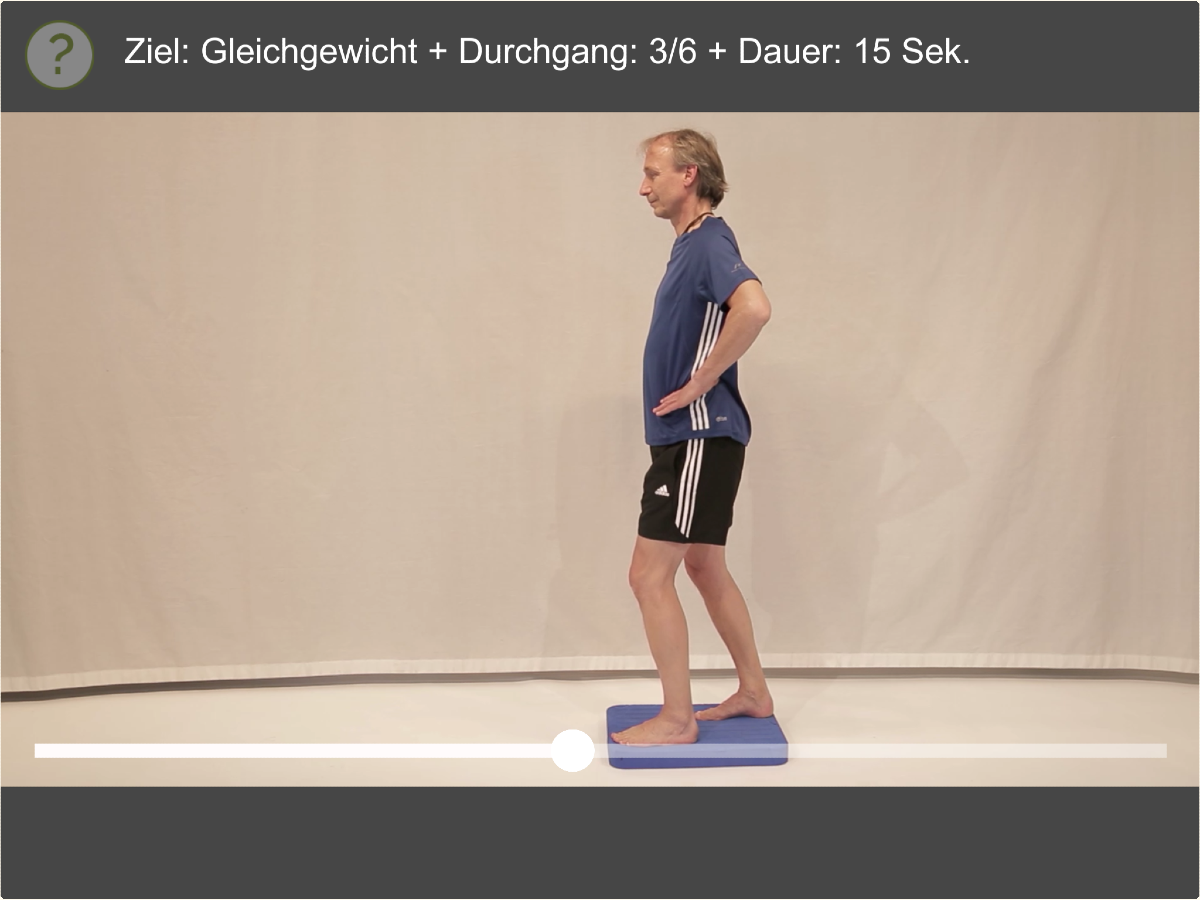

Supplement: Multimedia Appendix 4 [file jmir_v22i9e18233_app4.png]

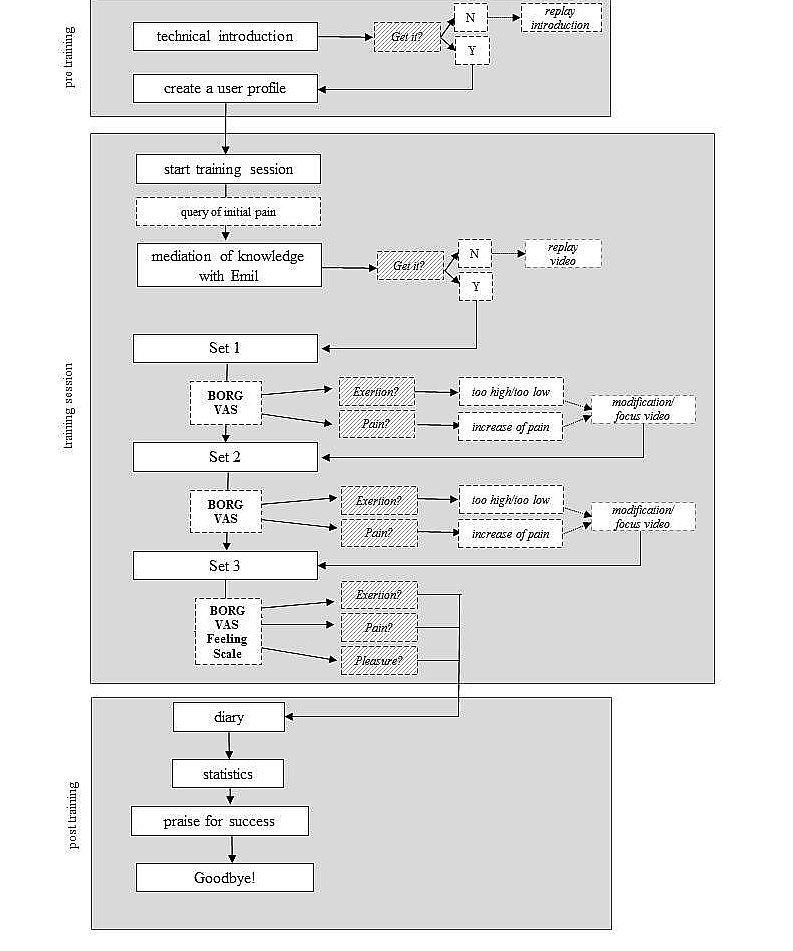

Supplement: Multimedia Appendix 6 [file jmir_v22i9e18233_app6.png]
