# Supplementary material for: Effectiveness of Human Versus Computer-Based Instructions for Exercise on Physical Activity–Related Health Competence in Patients with Hip Osteoarthritis: Randomized Noninferiority Crossover Trial
Source: J Med Internet Res. 2020 Sep 28;22(9):e18233. doi: 10.2196/18233 (PMC7551118; doi:10.2196/18233)
Supplement: Multimedia Appendix 5 [file jmir_v22i9e18233_app5.docx]

| App components and theoretical background of the interaction principles. | | |
| --- | --- | --- |
| **Components** | **Content and Function in App** | **Theoretical Background of Interaction Principles** |
| technical introduction | - familiar interaction with app - overcoming technical barriers and fears |  |
| user profile | - reference values, baseline characters  1. demographic data 2. complaints and problems with the hip joint [1] 3. PA 4. monthly exercise |  |
| pedagogical agent Emil   1. male human 2. athletic training partner 3. facial expressions 4. slight movements | - teaching knowledge  1. on training 2. on dealing with pain | - „persona effect“ of pedagogical agents   - cognitive effect in knowledge acquisition and motivational aspects [2,3] - human-likes animations (appearance)   - more intelligent and educational [4,5]   - greater user attention [4]   - entertainment and engagingness [4] - on-screen agent   - no effect on split-attention [6] - moving agents show better learning results than static ones [6]   - drawing attention to important elements |
| exercise introduction | - provide a set of exercises for perception, strength (abductors and hip extension), and coordination/balance - between each set: statement of the individual about exertion and pain | - principles of observational learning [7]   - combination of practice and observation and training in dyads [8-11]     - skills of model [11]   - model-observer similarity [12,13]   - different gender-model [14]   - skills of model   - feedback     - external focus [7,15]     - simultaneous [7,16]     - self-controlled [17-19] - guiding attentional focus of learners [20-28]   - Evoke external focus (acoustic support for motor execution (rhythm) in exercise videos) |
|  | - video-based instructions  1. instruction videos    - explains exercise in detail    - initial/final position 2. exercise videos    - supports during exercise    - model as personal training partner and visual corrective    - includes visual and auditive support 3. focus videos    - focus on initial position in case of too much pain 4. intensity adjustment    - explains how to decrease or to increase intensity |  |
| feedback-based dose adjustments and further instructions | - Depending on the exertion and pain, the instructions for the intensity of the further sets of the exercise is adjusted - individualize appropriate dose of exercise - foster task-specific knowledge and skills | - subject-based intensity regulation   - quantitating the intensity of training (RPE [29],VAS [30]) - positive experiences regarding exercise, e.g. motivational processes [31]   - change in affect (Feeling Scale [32]) |

1. Blasimann A, Dauphinee SW, Staal JB. Translation, Cross-cultural Adaptation, and Psychometric Properties of the German Version of the Hip Disability and Osteoarthritis Outcome Score. Journal of Orthopaedic & Sports Physical Therapy. 2014;44(12):989-97. PMID: 25394689. doi: 10.2519/jospt.2014.4994.

2. Dinçer S, Doğanay A. The effects of multiple-pedagogical agents on learners’ academic success, motivation, and cognitive load. Computers & Education. 2017 2017/08/01/;111:74-100. doi: https://doi.org/10.1016/j.compedu.2017.04.005.

3. Lester JC, Converse SA, Kahler SE, Todd Barlow S, Stone BA, Bhogal RS. The persona effect: affective impact of animated pedagogical agents. Proceedings of the ACM SIGCHI Conference on Human factors in computing systems; Atlanta, Georgia, USA. 258797: ACM; 1997. p. 359-66.

4. Dehn DM, Van Mulken S. The impact of animated interface agents: a review of empirical research. International Journal of Human-Computer Studies. 2000 2000/01/01/;52(1):1-22. doi: https://doi.org/10.1006/ijhc.1999.0325.

5. Kizilcec F, Papadopoulos K, Sritanyaratana L. Showing face in video instruction: effects on information retention, visual attention, and affect. Proceedings of the 32nd annual ACM conference on Human factors in computing systems; Toronto, Ontario, Canada. 2557207: ACM; 2014. p. 2095-102.

6. Craig S, Gholson B, M. Driscoll D. Animated pedagogical agents in multimedia educational environments: Effects of agent properties, picture features and redundancy. Journal of Educational Psychology. 2002;94:428-34. doi: 10.1037/0022-0663.94.2.428.

7. Wulf G. Motorisches Lernen - Therapierelevante Forschungsergebnisse. Ergoscience. 2007;2:47-55. doi: 10.1055/s-2007-963010.

8. Gulz A, Haake M. Design of animated pedagogical agents—A look at their look. International Journal of Human-Computer Studies. 2006 2006/04/01/;64(4):322-39. doi: https://doi.org/10.1016/j.ijhcs.2005.08.006.

9. Shea C, Wright D, Wulf G, Whitacre C. Physical and Observational Practice Afford Unique Learning Opportunities2000. 27-36 p.

10. Shebilskem WL, Regian JW, Arthur W, Jordan JA. A Dyadic Protocol for Training Complex Skills. Human Factors. 1992;34(3):369-74. doi: 10.1177/001872089203400309.

11. McCullagh P, Meyer K. Learning versus correct models: influence of model type on the learning of a free-weight squat lift. Research Quarterly for Exercise and Sport. 1997;68(1):56-61.

12. Schunk DH. Peer Models and Children’s Behavioral Change. Review of Educational Research. 1987;57(2):149-74. doi: 10.3102/00346543057002149.

13. Hoogerheide V, van Wermeskerken M, Loyens SMM, van Gog T. Learning from video modeling examples: Content kept equal, adults are more effective models than peers. Learning and Instruction. 2016 2016/08/01/;44:22-30. doi: https://doi.org/10.1016/j.learninstruc.2016.02.004.

14. Hoogerheide V, Loyens SMM, van Gog T. Learning from video modeling examples: does gender matter? Instructional Science. 2016 February 01;44(1):69-86. doi: 10.1007/s11251-015-9360-y.

15. Shea CH, Wulf G. Enhancing motor learning through external-focus instructions and feedback. Human Movement Science. 1999 1999/08/01/;18(4):553-71. doi: https://doi.org/10.1016/S0167-9457(99)00031-7.

16. Grand KF, Bruzi AT, Dyke FB, Godwin MM, Leiker AM, Thompson AG, et al. Why self-controlled feedback enhances motor learning: Answers from electroencephalography and indices of motivation. Human Movement Science. 2015 2015/10/01/;43:23-32. doi: https://doi.org/10.1016/j.humov.2015.06.013.

17. Winstein CJ, Pohl PS, Cardinale C, Green A, Scholtz L, Waters CS. Learning a Partial-Weight-Bearing Skill: Effectiveness of Two Forms of Feedback. Physical Therapy. 1996;76(9):985-93. doi: 10.1093/ptj/76.9.985.

18. Chiviacowsky S, Lessa H. Choices Over Feedback Enhance Motor Learning in Older Adults. 2017;5:304-18. doi: 10.1123/JMLD.2016-0031.

19. Lewthwaite R, Chiviacowsky S, Drews R, Wulf G. Choose to move: The motivational impact of autonomy support on motor learning. Psychonomic Bulletin & Review. 2015;22. doi: 10.3758/s13423-015-0814-7.

20. Wulf G. Attentional focus and motor learning: A review of 15 years. International Review of Sport and Exercise Psychology. 2013;6:77-104. doi: 10.1080/1750984X.2012.723728.

21. Wulf G, Lewthwaite R. Optimizing performance through intrinsic motivation and attention for learning: The OPTIMAL theory of motor learning. Psychonomic Bulletin & Review. 2016;23. doi: 10.3758/s13423-015-0999-9.

22. Lohse KR, Sherwood DE, Healy AF. How changing the focus of attention affects performance, kinematics, and electromyography in dart throwing. Human Movement Science. 2010 2010/08/01/;29(4):542-55. doi: https://doi.org/10.1016/j.humov.2010.05.001.

23. Lohse KR, Sherwood DE, Healy AF. On the advantage of an external focus of attention: A benefit to learning or performance? Human Movement Science. 2014 2014/02/01/;33:120-34. doi: https://doi.org/10.1016/j.humov.2013.07.022.

24. Chua L-K, Wulf G, Lewthwaite R. Onward and upward: Optimizing motor performance. Human Movement Science. 2018;60. doi: 10.1016/j.humov.2018.05.006.

25. Kal EC, van der Kamp J, Houdijk H. External attentional focus enhances movement automatization: A comprehensive test of the constrained action hypothesis. Human Movement Science. 2013 2013/08/01/;32(4):527-39. doi: https://doi.org/10.1016/j.humov.2013.04.001.

26. Zachry T, Wulf G, Mercer J, Bezodis N. Increased movement accuracy and reduced EMG activity as the result of adopting an external focus of attention. Brain Research Bulletin. 2005 2005/10/30/;67(4):304-9. doi: https://doi.org/10.1016/j.brainresbull.2005.06.035.

27. Krishnappa Ramamoorthy RP, Vadhel V, Sunderraj Pandian J, Jain M. Influence of focus of attention on motor learning among elderly—a randomized control trial. Physiotherapy. 2015;101:e793-e4. doi: 10.1016/j.physio.2015.03.3674.

28. Land WM, Frank C, Schack T. The influence of attentional focus on the development of skill representation in a complex action. Psychology of Sport and Exercise. 2014 2014/01/01/;15(1):30-8. doi: https://doi.org/10.1016/j.psychsport.2013.09.006.

29. Sweet TW, Foster C, McGuigan MR, Brice G. Quantification of restistance training using the session RPE method. Journal of Strength and Conditioning Research. 2004;18(4):796-802.

30. Hawker G, Mian S, Kendzerska T, French M. Measures of adult pain: visual analog scale for pain (VAS Pain), numeric rating scale for pain (NRS Pain), McGill Pain Questionnaire (MPQ), Short-Form McGill Pain Questionnaire (SF-MPQ), Chronic Pain Grade Scale (CPGS), Short Form-36 bodily pain scale (SF-36 BPS), and Measure of Intermittent and Constant Osteoarthritis Pain (ICOAP). Arthritis care & research. 2011 11/01;63 S240-52. doi: 10.1002/acr.20543.

31. Sudeck G, Schmid J, Conzelmann A. Exercise experiences and changes in affective attitude: Direct and indirect effects of in situ measurements of exercise. Frontiers in Psychology. 2016 2016;7:900. doi: 10.3389/fpsyg.2016.00900.

32. Hardy CJ, Rejeski WJ. Not what, but how one feels: The measurement of affect during exercise. Journal of Sport & Exercise Psychology. 1989 1989;11:304-17.
