# Supplementary material for: Effectiveness of Human Versus Computer-Based Instructions for Exercise on Physical Activity–Related Health Competence in Patients with Hip Osteoarthritis: Randomized Noninferiority Crossover Trial
Source: J Med Internet Res. 2020 Sep 28;22(9):e18233. doi: 10.2196/18233 (PMC7551118; doi:10.2196/18233)
Supplement: Multimedia Appendix 7 [file jmir_v22i9e18233_app7.docx]

| Appendix 7: Rater agreement for movement quality, averaged across all categories and sets for each exercise and intervention in percent. Physiotherapist (P), App (A). | | |
| --- | --- | --- |
| **Exercise** | **P** | **A** |
| **MQ_mobility_seated (%)** | 83 | 84 |
| **MQ_strength_supine (%)** | 91 | 87 |
| **MQ_strength_table (%)** | 84 | 79 |
| **MQ_balance_stance (%)** | 84 | 81 |
