# Supplementary material for: Effectiveness of Human Versus Computer-Based Instructions for Exercise on Physical Activity–Related Health Competence in Patients with Hip Osteoarthritis: Randomized Noninferiority Crossover Trial
Source: J Med Internet Res. 2020 Sep 28;22(9):e18233. doi: 10.2196/18233 (PMC7551118; doi:10.2196/18233)
Supplement: Multimedia Appendix 8 [file jmir_v22i9e18233_app8.docx]

**Appendix 8: Sensitivity Analysis for Movement Quality after Excluding Categories with Poor Interrater Reliability.**

Agreement between raters was not satisfactory in all cases. First, an in-depth analysis was undertaken to find out to what extent raters did not agree: by calculating the differences in point score for each rating between raters, ranging from 2 (total disagreement) along 1 (partial disagreement) to 0 (agreement).

Subsequently, all categories went through the following decision tree to decide whether to include categories in the sensitivity analysis for movement quality or not. The categories were deleted if:

- 1st decision: Agreement of categories Rater 1 vs. Rater 2 <75% (per category and set)
- 2nd decision: More than five cases in a category have a deviation of 2 points.
- 3rd decision: If a set still has a lower match than 60%, the category is eliminated.

After these elaborate considerations, the following numbers of categories were removed from the rating sheet: MQ_mobility_seated_sel: 3 of 12 categories; MQ_strength_supine_sel 3 of 19 categories; MQ_strength_table_sel: 9 of 21 categories and MQ_balance_stance_sel: 4 of 13 categories. For the selected categories, a mixed-model ANOVA was used for a renewed analysis. The results depicted in Table Appendix 8 show that MQ increased. However, analysis of the selected categories is comparable and the statistical statement remains the same.

| Table Appendix 8: Results (ANOVA mixed model) of selected categories (_sel). Physiotherapist (P), App (A). Estimated mean (emmean), standard error (SE) π: period effect, λ: carry-over effect and treatment effect differences (τd, 95% confidence interval CI), averaged over the levels of period and sequence. ^a^Hedges g_IG_: effect size (95% CI). ^b^Positive values indicate beneficial effect for P. Non-inferiority (ni) App (“1” if g_IG_ +95% CI <0.2, else “0”). ^c^Movement quality (MQ) with 0-100% of quality criteria points fulfilled. | | | | | | | |
| --- | --- | --- | --- | --- | --- | --- | --- |
|  | emmean (95% CI) | | ANOVA mixed model (α=0.05) | | | Effect size^a^ |  |
| Variable | P | A | π  (*P*) | λ  (*P*) | τ_d_  (95% CI)^b^ | g_IG_  (95% CI) | ni |
| **Movement Quality sel (MQ)^c^** | **n=54** | |  |  |  |  | |
| 1° MQ_overall_sel | 93.5  (92.7, 94.4) | 91.9  (91.0, 92.7) | .003 | .78 | 1.66  (0.72, 2.60) | 0.52  (0.22, 0.84) | 0 |
| 2° MQ_mobility_seated_sel | 84.3  (82.0, 86.7) | 85.0  (82.7, 87.4) | .01 | .24 | -0.72  (-3.18, 1.75) | -0.09  (0.36, 0.19) | 1 |
| 2° MQ_strength_supine_sel | 96.5  (95.4, 97.6) | 92.8  (91.8, 93.9) | .92 | .82 | 3.70  (2.26, 5.13) | 0.96  (0.55, 1.37) | 0 |
| 2° MQ_strength_table_sel | 97.0  (95.8, 98.2) | 94.3  (93.1, 95.5) | .01 | .04 | 2.77  (1.30, 4.14) | 0.62  (0.28, 0.96) | 0 |
| 2° MQ_balance_stance_sel | 96.2  (95.1, 97.3) | 95.5  (94.4, 96.5) | .15 | .20 | 0.78  (-0.61, 2.17) | 0.20  (0.14, 0.54) | 0 |
